# Smartphone Abuse Amongst Adolescents: The Role of Impulsivity and Sensation Seeking

**DOI:** 10.3389/fpsyg.2021.746626

**Published:** 2021-12-16

**Authors:** Gloria Pérez de Albéniz Garrote, Laura Rubio, Begoña Medina Gómez, Cristina Buedo-Guirado

**Affiliations:** ^1^Departamento de Ciencias de la Educación, Universidad de Burgos, Burgos, Spain; ^2^Departamento de Psicología Evolutiva y de la Educación, Universidad de Granada, Granada, Spain; ^3^Universidad Internacional de la Rioja, Logroño, Spain

**Keywords:** adolescents, impulsivity, sensation-seeking, mobile phone abuse, dysfunctional impulsivity

## Abstract

Adolescence is the stage of development where the reward and emotional regulation systems are yet to be adjusted and where most excessive behaviors start, like smartphone abuse. In addition, in this evolutionary period adolescents are more susceptible to behavioral changes through specific interventions or educational programs. Thus, it is fundamental to analyze the personality profile of those adolescents showing excessive mobile phone usage to properly approach later prevention strategies. Impulsivity is one of the most repeated variables associated with teenage addictions, although it has been observed that not all impulsive behaviors need to be detrimental. The aim of this study is to analyze how impulsivity affects smartphone addiction directly, but also indirectly, by assessing its association with sensation seeking variables (thrill and adventure seeking, experience seeking, disinhibition, and boredom susceptibility) which are in turn decisive when using these technologies improperly. The sample was made up of 614 adolescents aged 13–18 attending secondary education from Burgos, Spain. Dickman Impulsivity Inventory, Sensation Seeking Scale, and Ad-hoc questionnaire on adolescent self-perception as to smartphone use were applied. Results show that 41.4% of participants admit to abusing smartphones sometimes, while 18.3% admit to abusing them more frequently and 24% to, at least ever, having defined themselves as smartphone addicts. Stepwise regression analysis revealed that gender (female), dysfunctional impulsivity and sensation seeking (disinhibition and thrill and adventure seeking) evidence 15.7% of variance in smartphone abuse. In addition, sensation seeking (thrill and adventure seeking, disinhibition, and boredom susceptibility) were found to mediate the relationship between dysfunctional impulsivity and smartphone abuse. Therefore, dysfunctional impulsivity was directly connected with teenage smartphone abuse, but also had an indirect stronger association through thrill and adventure seeking, disinhibition and boredom susceptibility.

## Introduction

The relationship between adolescents and their way to use information and communication technologies (hereinafter referred to as ICTs) have raised much interest in research over the last few years, in view of the number of publications in this respect. ICTs have involved great benefits in labor, education and society, but all its advantages are eclipsed when used improperly, since they may generate psychophysiological, affective and social problems.

There is no consensus in the scientific literature about the best term to refer to improperly use of the smartphone; the most commonly used concepts are problematic, abusive, excessive or maladaptive use. However, all of them refer to excessive use in time, inappropriate in situation and that interferes with the person’s daily activities at a personal, work, academic level and/or in relationships with family or friends (De Sola-Gutiérrez et al., 2013; Bian and Leung, 2015; Aljomaa et al., 2016; Panova and Carbonell, 2018). Nevertheless, the limits of abusive behavior are blurred, for example, for Montag and Walla (2016) the relationship between smartphone use and adaptive functioning represents an inverted U-curve, where moderate use has beneficial effects but excessive use has harmful consequences.

The potentially negative effects of an excessive use of the smartphone become especially important amongst teenagers, since the psychological skills which support their decision-making and moderate risk-taking are still developing (Steinberg, 2005). The adolescent brain has not yet completed its development, especially the prefrontal areas, which are involved in processes of regulation, planning and execution of action, so they are more vulnerable to abusive behaviors (Prencipe et al., 2011). In addition, some authors link lower inhibitory control to problematic ICT use in adolescents (Marco and Chóliz, 2012; Pedrero-Pérez et al., 2021). Thus, adolescence is the stage at which most excessive behaviors start, as occurs, for example, with alcohol consumption (Zych et al., 2020).

Other research lines suggest that there are gender differences in smartphone abuse behavior and that women are more at risk of misusing this technological support (Rial et al., 2015; Álvarez and Moral, 2020; Li et al., 2021), although further research in this direction is still needed.

Even when spending too much time on the mobile phone does not make an adolescent an addict, different studies relate excessive smartphone usage to mental health issues. In this way, it has been related to stress, social anxiety and depression (Lee et al., 2014; Seo et al., 2016; Lapierre et al., 2019), low self-esteem, cognitive and academic performance issues (Caplan, 2007; Leung, 2008; Marín-Vila et al., 2018), substance abuse (Sánchez-Martínez and Otero, 2009; López-Fernández et al., 2012) and Fear of Missing Out (FoMO) (Alt, 2015; Santana-Vega et al., 2018; Wolniewicz et al., 2018), especially at this stage of development where the sense of belonging plays such an important part.

Although it is complicated to determine the directionality of the relationship between smartphone abuse and mental health problems, it can indeed be affirmed that adolescents who displays excessive use of their smartphone often do so as a strategy to cope with negative emotions (Sun et al., 2019), for example boredom, social anxiety and depression (Yue et al., 2021).

In any case, if the abusive use of smartphone precedes mental suffering, dependence on such devices can be reduced to maintain or improve wellbeing. For this purpose, it is fundamental to analyze the personality profile of those young adults that show excessive mobile phone usage and impulsive behavior turns out to be one of the most recurring variables when delving into teenage addiction predisposition. In fact, impulsive behavior is considered one of the most relevant vulnerability axes (Billieux et al., 2007, 2008; Andreassen et al., 2013; Roberts et al., 2015).

However, not all impulsive behaviors need to be problematic, especially in tasks which need to be completed in a short period of time. For such a reason, some researchers have suggested two types of impulsivity: functional and dysfunctional (Dickman, 1990, 1993, 2000). Functional impulsivity is a quick information processing model which usually leads to correct answers, while dysfunctional impulsivity is a tendency to make quick, poorly planned and impulsive decisions which could result in mistakes or problems (Pedrero-Pérez et al., 2012).

Dysfunctional impulsive behaviors may increase adolescents’ likelihood to adopt behaviors which put their health at risk, such as alcohol abuse (Narváez and Caro, 2015), predisposition to start or increase cannabis consumption (Moreno et al., 2012; Limonero et al., 2013) or an excessive usage of internet and smartphones (Billieux et al., 2008; Moral and Fernández, 2019). Later on, impulsive behaviors are maintained by positive reinforcement and are usually oriented toward the achievement of a hedonic goal (Pedrero-Pérez et al., 2021).

As to the sensation seeking variable, it may be categorized as a psychobiological disposition featured by the search for varied, novel, complex and intense experiences and feelings, as well as a by a certain inclination to become involved in risky situations (Zuckerman, 2007). Although it is true that, ontogenetically speaking, this personality feature is more remarkable in adolescence than at other developmental stages, certain variations can be appreciated from an individual to another.

Sensation seeking has been linked to risky behaviors in adolescence. For example, it has been related to smoking (Latorre-Román et al., 2014), an excessive consumption of alcohol and other substances (Zuckerman and Aluja, 2014; Merchán et al., 2020) and smartphone addiction (Leung, 2008). Especially linked to risk-taking are the disinhibition and the thrill and adventure seeking subscales of the *Zuckerman Sensation Seeking questionnaire* (Lac and Donaldson, 2021; Siraj et al., 2021), both related to impulsive behavior as well (Stoyanova and Ivantchev, 2021).

Thus, impulsivity and sensation seeking are two variables directly associated with abusive behavior, for example with smartphone use. In addition, impulsivity, especially dysfunctional, is directly related to sensation seeking, and may have an essential role in excessive behaviors. In fact, many researchers show that sensation seeking, functional and dysfunctional impulsivity had a significant and positive correlation (Magid et al., 2007). Moreover, dysfunctional impulsivity seems to be the one which shows a more direct impact on sensation seeking (Stoyanova and Ivantchev, 2021), which may turn the variable into a mediator between impulsivity and excessive behaviors.

This relationship between both variables has an exceptionally explanatory value (Myrseth et al., 2012; De Sola-Gutiérrez et al., 2013). Being highly sensitive to new stimuli involves exploring and trying new experiences. It is therefore the presence of impulsivity as associated with sensation seeking which may precede an abusive behavior.

Consequently, the aim of this research was, on the one hand to analyze the direct association of impulsivity and sensation seeking scales (thrill and adventure seeking, experience seeking, and disinhibition and boredom susceptibility) on smartphone usage; and, on the second hand to evaluate the indirect association of dysfunctional impulsivity on smartphone abuse, through the sensation seeking variables which are in turn decisive when using these technologies improperly. Thus, greater dysfunctional impulsivity increases the variables related to sensation seeking, and this ultimately increases the smartphone abuse.

## Materials and Methods

### Procedure

In the first place, the aims and purposes of this study were presented to the Provincial Department of Education in Burgos, Spain. Once the permissions were obtained, eight public educative centers were selected using random cluster sampling to conduct the study. Each center was informed about the study and their voluntary participation was requested. The tests were applied on all the students between 13 and 18 years who voluntarily agreed to participate, after informed consent was obtained from their parents. The inclusion criteria did not require having a smartphone to participate in the study. Tools used in this study were administered collectively in a classroom with the presence of their class teacher and a member of the research team. Participants were informed again that participation was voluntary, that the responses were anonymous and that they could withdraw from participating at any time. Then, each student answered individually and anonymously in 20 mins approximately.

### Tools Used

#### Dickman Impulsivity Inventory

The DII (Dickman, 1990; Chico et al., 2003) is a 23-item true/false questionnaire divided into two subscales: (a) Functional impulsivity (FI) with 11 items (i.e.: “I am uncomfortable when I have to make up my mind rapidly”), and (b) Dysfunctional impulsivity (DI) with 12 items (i.e.: “I will often say whatever comes into my head without thinking first”). The Spanish version of the instrument (Chico et al., 2003) has been used frequently in adolescent samples and shows adequate psychometric properties (Vigil-Coleṭ and Morales-Vives, 2005; Vigil-Colet et al., 2008; Puerta-Cortés et al., 2017). Cronbach’s alpha values in Spanish version were 0.74 for functional impulsivity and 0.86 for dysfunctional impulsivity (Pedrero-Pérez, 2009).

#### Sensation Seeking Scale V

This scale (Zuckerman et al., 1978; Tous, 1984) consists of 40 true/false items divided into four subscales of 10 items each: (a) Thrill and adventure seeking (TAS), that refers to the desire to get involved in extreme activities by the unusual sensations they cause (i.e.: “I often wish I could be a mountain climber”); (b) Experience seeking (ES), that refers to the search for activation from unconventional lifestyles: experiences through the senses, travel, art, music, food, clothing, the style of bohemian life and the company of unconventional friendships (i.e.: “I like to explore a strange city or section of town myself, even if it means getting lost”); (c) Disinhibition (Dis), that reflects the desire to experiment through sexual and social stimulation, fun and parties (i.e.: “I like wild “uninhibited” parties”); and (d) Boredom susceptibility (BS) which refers to intolerance to monotonous and predictable conditions (i.e.: “I can’t stand watching a movie that I’ve seen before”). This questionnaire has often been used in adolescent samples showing good psychometric properties (Martínez-Lorca and Alonso-Sanz, 2003; Padrós Blázquez et al., 2020). The Spanish version of the instrument (Chico, 2000) showed an adequate reliability of the global scale (Cronbach’s alpha ranged between 0.83 and 0.86), and for four subscales Cronbach’s alpha between 0.56 and 0.82.

#### Ad-hoc Questionnaire on Adolescent Self-Perception as to Smartphone Use

This instrument was built for the purpose of this research with the aim to evaluate the habits of the participants in relation to the use of the telephone and possible consequences of inappropriate use. Ad-hoc questionnaire consists of nine questions with five possible answers (1 = never, 2 = sometimes, 3 = often, 4 = nearly always, 5 = always). The questions were related to have a smartphone, how often they changed mobile phone, to have discussions with parents about using the phone, if parents felt that their children were spending a lot of money on the smartphone, influence of the use of smartphone in school performance, anger or irritation from not using the phone, if they could live without a smartphone, if they felt they were abusing the phone and if they considered themselves addicted. The question about whether they felt they were abusing the smartphone was used as a dependent variable in the analyzes carried out.

### Ethics Statement

The ethical standards of the American Psychological Association (APA) were employed to design the research and data collection.

### Statistical Analysis

Data analysis was performed with SPSS-25 statistical package, using frequencies to describe the percentage of use and abuse of smartphone, and stepwise regression to assess factors which could influence smartphone abuse, evaluated by asking if they feel they abuse of the smartphone. Factors included were gender, age, sensation seeking scales and functional and dysfunctional impulsivity.

**FIGURE 1:**
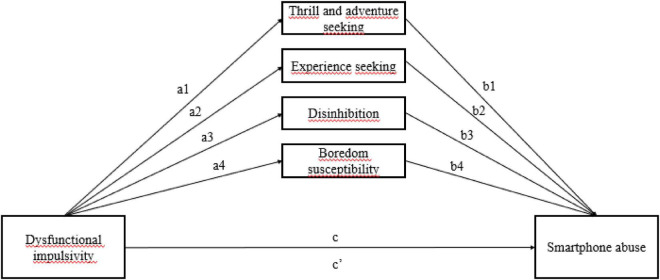
Proposed model of the indirect effect of dysfunctional impulsivity on smartphone abuse through sensation seeking scales.

In order to evaluate the mediation of sensation seeking in the relationship between dysfunctional impulsivity and smartphone abuse (see Figure 1), a multiple mediation analysis was performed with PROCESS tool using the bias-corrected bootstrapping method (Preacher and Hayes, 2004, 2008). Percentile bootstrap confidence intervals (CIs) are examined to determine significative paths, if zero is contained within the 95% CIs, then the lack of significance is assumed (Shrout and Bolger, 2002; Preacher and Hayes, 2008).

### Sample and Participants

The sample was comprised of 614 adolescents, 49% male and 51% female, aged 13–18 years (*M* = 15.76, *SD* = 1.16), attending secondary education in eight publics or privates schools of Burgos, Spain.

Regarding the educational level, all participants were enrolled in secondary education. Specifically, 22.5% were in the 3rd year of ESO (Compulsory Secondary Education), 26.9% in the 4th year of ESO, 27% in the 1st year of Spanish baccalaureate and 23.6% were studying in the 2nd year. Regarding living conditions, most of the participants lived with their parents (86%), 11% lived only with their mother, 2% with their father, and 0.6% with another family member.

## Results

### Results of the General Smartphone Use

With regard to the use made of the smartphone, majority of participants have one of their own (98.5%), and 30.3% of them change phones every year and 50.7% every 2 years. A total of 25.2% of participants admit that they have fought with their parents due to the use they make of the mobile phone, 41.8% state that their parents think that they spend too much money on mobile phones and 15.8% consider that mobile use might have reduced their achievement at school. More than a third of the participants, 37.7%, have, at least sometimes, felt anxious or irritated when unable to use mobile phones and only 32.1% stated that they could easily do without them. Moreover, 41.4% admit to abusing smartphones sometimes, while 18.3% admit to abusing them more frequently and 24% to, at least ever, having defined themselves as smartphone addicts.

### Results of the Stepwise Regression Analysis

In order to determine the factors associated with smartphone addiction in adolescents, a stepwise regression analysis was performed. Gender, age, sensation seeking variables (thrill and adventure seeking, experience seeking, and disinhibition and boredom susceptibility), functional and dysfunctional impulsivity were included as factors in the analysis. As Table 1 conveys, results of the analysis show that gender (being female), dysfunctional impulsivity and sensation seeking (disinhibition and thrill and adventure seeking) were predictors that explained, in total, 15.8% of variance in smartphone abuse. Nevertheless, age, experience seeking, boredom susceptibility and functional impulsivity were not significant factors related to smartphone abuse.

**TABLE 1:** Results of the stepwise regression analysis for smartphone abuse.

Step	Variable	*B*	β	*R*	*R* ^2^	Corrected *R*^2^	Change in *R*^2^	*p*
1	Gender	0.429	0.269	0.269	0.073	0.071	0.073	0.000
2	Disinhibition	0.076	0.232	0.355	0.126	0.123	0.054	0.000
3	Dysfunctional impulsivity	0.052	0.137	0.380	0.144	0.140	0.018	0.001
4	Thrill and adventure seeking	−0.034	−0.123	0.398	0.158	0.152	0.014	0.003

### Multiple Mediation Analysis of Sensation Seeking Scales

The mediation analysis of sensation seeking variables in the relationship between dysfunctional impulsivity and smartphone abuse was analyzed using the 5,000 bootstrap and bias-corrected and accelerated 95% CI procedure (Preacher and Hayes, 2004). Once gender had been controlled for, results revealed that paths a, from dysfunctional impulsivity to sensation seeking scales were significant (see Table 2). On the other hand, with regards to the paths b, from the proposed mediators to mobile abuse, three of four paths were significant. Specifically, thrill and adventure seeking, disinhibition and boredom susceptibility as Table 2 conveys.

**TABLE 2:** Indirect effect of dysfunctional impulsivity on smartphone abuse through sensation seeking scales.

Mediator	Effect of X on M (a)	SE	Effect of M on Y (b)	SE	Bootstrap estimate	SE	BCa 95% CI	
							Lower	Upper
Thrill and adventure seeking	0.01622**	0.0591	−0.0420***	0.0125	–0.0068	0.0032	–0.140	–0.0015
Experience seeking	0.1630***	0.0425	0.0096	0.0204	0.0016	0.0035	–0.0052	0.0089
Disinhibition	0.2070***	0.0507	0.0676***	0.0166	0.0140	0.0051	0.0055	0.0251
Boredom susceptibility	0.2564***	0.0516	0.0331*	0.0142	0.0085	0.0041	0.0011	0.0172

*Based on 5,000 bootstrap samples.*

*BCa, bias corrected and accelerated; CI, confidence interval.*

*p < 0.05; **p < 0.01; ***p < 0.001.

In addition, when controlling for gender (*B* = 0.4129, *SE* = 0.0684, *p* < 0.001), the total effect (path c) of dysfunctional impulsivity on smartphone addiction was significant (total effect: *B* = 0.0664, *SE* = 0.0162, *p* = 0.001) and higher than the direct effect (path c′) (direct effect: *B* = 0.0514., *SE* = 0.0162, *p* < 0.05) The proposed model explained 9.31% (*F* = 28.7891, *p* < 0.001) of the variance of mobile phone addiction.

Total indirect effect of dysfunctional impulsivity on mobile addiction through the sensation seeking variables was significant (*B* = 0.0184, at 95% confidence lower limit = 0.0049, upper limit = 0.0337). Regarding to the specific indirect effect (see Table 2), three of the proposed mediators were significant: thrill and adventure seeking (*B* = −0.0068, at 95% confidence lower limit = −0.0140, upper limit = −0.0015), disinhibition (*B* = 0.0140, at 95% confidence lower limit = 0.0055, upper limit = 0.0251) and boredom susceptibility (*B* = 0.0085, at 95% confidence lower limit = 0.0011, upper limit = 0.0172).

## Discussion

This study is intended to give response to some unanswered questions on the personality variables related to smartphone abuse in adolescents, as well as determining some direct and indirect factors related to unadaptive smartphone usage.

Specifically, the aim of this research was, on the one hand to analyze the smartphone use among adolescents between the ages of 13 and 18; to assess the direct association of impulsivity and sensation seeking scales (thrill and adventure seeking, experience seeking, and disinhibition and boredom susceptibility) on smartphone abuse; and, on the other hand to evaluate the indirect association of dysfunctional impulsivity on smartphone abuse, through the sensation seeking variables.

Results show that smartphone use and abuse by this adolescent sample was high. Specifically, a 41.4% of participants admit to abusing smartphones sometimes, while 18.3% admit to abusing them more frequently and 24% have ever defined themselves as smartphone addicts. Stepwise regression analysis revealed that gender (female), dysfunctional impulsivity and sensation seeking (disinhibition and thrill and adventure seeking) evidence 15.7% of variance in smartphone abuse. In addition, sensation seeking (thrill and adventure seeking and disinhibition and boredom susceptibility) were found to mediate the relationship between dysfunctional impulsivity and smartphone abuse.

Use and abuse of ICTs and, in particular, of smartphones, has increased rapidly over the last few years. Results show a high usage of smartphones amongst the adolescents of this sample. Virtually all of them have their own smartphone and keep switching to new models. Moreover, a third of them declare that being unable to use the smartphone cause negative feelings in them, such as anger. Also, almost half of the respondents affirmed that they could be considered mobile abusers in some way, while a fourth part define themselves as mobile addicts. Prior studies conducted on Spanish population showed that approximately a quarter of the population could be considered dependent on mobile phones (Sánchez-Martínez and Otero, 2009; Ballestar-Tarín et al., 2020) and that the younger the population was, the more dependent they were (Ballestar-Tarín et al., 2020). Similar results have been observed in other cultural environments (Kwon et al., 2013; Machado-Khoury et al., 2019). These data are quite significant, since they demonstrate the excessive use which is often made of smartphones amongst young adults. However, in the present study, age was not a significant factor in smartphone abuse, so more research would be needed in this regard in the Spanish adolescent population.

On the other hand, by analyzing factors intimately related to excessive smartphone usage, several significant variables were observed. Being a female was associated with greater smartphone addiction, apart from dysfunctional impulsivity and two of the sensation seeking scales (disinhibition and thrill and adventure seeking).

As to gender, this is a controverted matter, as some authors consider being a female as a risk factor toward improper usage of smartphones (Rial et al., 2015; Álvarez and Moral, 2020; Li et al., 2021), with a greater dependence on them amongst females (Sánchez-Martínez and Otero, 2009; Álvarez and Moral, 2020; Ballestar-Tarín et al., 2020), while others do not find such a relation (Son et al., 2021). Our study shows a clearly direct relationship, as it is the first factor which emerges upon analysis of smartphone addiction and which itself explains 7.3% of improper mobile usage. Some authors affirm that female propensity to this sort of dependence is linked to their inclination to display more prosocial behavior (Veissière and Stendel, 2018). Another relevant aspect contemplated under the scientific literature is that, in some occasions, females state that using a smartphone help them face unpleasant mood or relieve emotional distress (Chóliz et al., 2009; García-Oliva and Piqueras, 2016).

As to impulsivity, results show that dysfunctional impulsivity was the third factor related to smartphone addiction, explaining a supplementary 1.8% of variance in this excessive behavior. These results show themselves consistent with those of other researchers relating impulsivity to predisposition to, or maintenance of, abusive behaviors toward technologies (Billieux et al., 2008; Meerkerk et al., 2010; Jiang and Shi, 2016). As discussed above, dysfunctional impulsivity is related to poor planning of own behaviors, which can lead to action without evaluating the consequences, and may facilitate smartphone dependence (Verdejo et al., 2008; Pedrero-Pérez, 2009; Kim et al., 2016).

We also observed a direct relation of sensation seeking variables on smartphone abuse in the present study, specifically disinhibition, which was the second associated factor, while thrill and adventure seeking was the fourth one, both explaining 5.4 and 1.4% of mobile phone addiction, respectively. Previous studies have approached smartphone addiction as associated with sensation seeking, specifically with boredom during leisure time and thrill and adventure seeking (Leung, 2008) and also with disinhibition, even when the latter was more linked to risk behaviors with a sexual intent (Schoeps et al., 2020). Disinhibition and thrill and adventure seeking seem to be the variables which are most associated with risk behaviors (Lac and Donaldson, 2021; Siraj et al., 2021). Thus, both factors seemingly relate to impulsive behavior in adolescence as well (Stoyanova and Ivantchev, 2021). In fact, sensation seeking is featured by behaviors that involve a risk or bring new thrills (Zuckerman, 2007), so it is logical that these variables are related to smartphone addiction, since this device provides quick access to apps and social media that provide new stimuli on a continuous basis.

Lastly, as has been observed, dysfunctional impulsivity has a direct association with teenage smartphone abuse, but also an indirectly stronger relationship through sensation seeking (thrill and adventure seeking and disinhibition and boredom susceptibility).

We can affirm that dysfunctional impulsivity is a vulnerability marker in smartphone abuse, especially when is linked to the sensation seeking variable (Beard, 2011; Dalbudak et al., 2013). Even Zuckerman himself, who was interested in boredom susceptibility in particular, later suggested a more comprehensive and psychobiology-based approach to personality, including a new dimension: impulsive sensation seeking, with an emphasis on the relationship between both variables (Zuckerman, 2006).

In this study, we appreciate that indirect relationship of impulsivity with smartphone abuse through sensation seeking becomes more important than the direct one. As a result, adolescents with boredom susceptibility, with disinhibited behavior and prone to thrill and adventure seeking that are also dysfunctionally impulsive may show an aggravated abuse of smartphones through a negative reinforcement process to soothe a dysphoric mood. Previous studies had already shown that abusive behaviors could be related to sensation seeking (boredom susceptibility, disinhibition and thrill and adventure seeking) (Leung, 2008; Lac and Donaldson, 2021; Siraj et al., 2021), but also that sensation seeking was related to dysfunctional impulsivity (Stoyanova and Ivantchev, 2021). Therefore, it is possible that dysfunctional impulsivity acts by increasing abusive behavior with smartphones as a consequence of the increased susceptibility of adolescents to sensation seeking. Nevertheless, experience seeking was not a mediator in the relationship between dysfunctional impulsivity and smartphone abuse. Previous studies have shown that experience seeking is not usually related to smartphone abuse or other abusive behaviors (Annalakshmi et al., 2020; Lac and Donaldson, 2021; Siraj et al., 2021), it is possible that for adolescents the smartphone is not seen as a way to get new experiences, and for that purpose they use video games, travel and other activities (Zuckerman and Aluja, 2014).

This study has some limitations which are worth mentioning. Some limitations are the ones related to the use of self-reports and which are associated with response validity, which may be quite subjective and subject to the participants’ honesty and the moment at which the tests are completed. Although the sample size is large, this study, just like most studies conducted in this line of research, is cross-sectional, hence the difficulties involved in establishing predictive effects and the need to verify the results in longitudinal studies. Future research should also delve into age effects. Some studies link higher impulsivity scores with a younger age, although this result was not observed in this study. It is important to know how the impulsivity and sensation seeking pattern are related to age in order to be able to design educational strategies as early as possible in order to avoid the development of abusive behaviors in young people.

Despite the above, it is important to conduct this sort of research to polish the interventions addressed to the development of a culture of healthy usage of smartphones in the familial and social spheres. In this way, this analysis of the different scales within sensation seeking in association with impulsive behavior will help us elaborate a more accurate profile of adolescents with an abusive behavior in the use of smartphones. As a result, we would manage to raise social awareness of the risk involved in smartphone abuse and its adverse effects on adolescence.

It is also important for the study to be focused on adolescence, as many works on smartphone addiction are based on samples above 18 years old. Adolescence is a particularly susceptible development stage where excessive behaviors can be associated with psychosocial and emotional development, with long-term consequences that can be seen in adulthood. However, in adolescence, it is easier for behavior to be changed through specific intervention or educational programs, hence the importance of knowing what profiles are linked to excessive behaviors. This study then shows that it is necessary to pay attention to an impulsive psychosocial profile with a susceptibility to sensation seeking (thrill and adventure seeking and disinhibition and boredom susceptibility), as these are indicative of a propensity to smartphone abuse.

## Data Availability Statement

The original contributions presented in the study are included in the article/supplementary material, further inquiries can be directed to the corresponding author.

## Ethics Statement

Ethical review and approval was not required for the study on human participants in accordance with the local legislation and institutional requirements. Written informed consent to participate in this study was provided by the participants’ legal guardian/next of kin.

## Author Contributions

GP formulated the research questions, designed the study, supervised the data collection, and wrote the manuscript. LR carried out the statistical analysis and wrote the manuscript. BM and CB were responsible for the statistical design of the study and assisted to wrote and edited the manuscript. All authors contributed to the article and approved the submitted version.

## Conflict of Interest

The authors declare that the research was conducted in the absence of any commercial or financial relationships that could be construed as a potential conflict of interest.

## Publisher’s Note

All claims expressed in this article are solely those of the authors and do not necessarily represent those of their affiliated organizations, or those of the publisher, the editors and the reviewers. Any product that may be evaluated in this article, or claim that may be made by its manufacturer, is not guaranteed or endorsed by the publisher.
